# Atraumatic splenic rupture in a child with COVID 19

**DOI:** 10.1186/s12887-022-03353-8

**Published:** 2022-05-21

**Authors:** Ilirjana Bakalli, Marsela Biqiku, Durim Cela, Adnand Demrozi, Ermira Kola, Ermela Celaj, Inva Gjeta, Durim Sala, Dea Klironomi

**Affiliations:** 1 PICU, UHC “Mother Theresa”, Tirane, Albania; 2 Department of Radiology, UHC “Mother Theresa”, Tirane, Albania; 3 Medical University of Vienna, Vienna, Austria

**Keywords:** Atraumatic, Splenic rupture, COVID-19, Children

## Abstract

**Background:**

The majority of children with COVID-19 have only minor symptoms or none at all. COVID-19, on the other hand, can cause serious illness in some children, necessitating hospitalization, intensive care, and invasive ventilation. Many studies have revealed that SARS-CoV-2 affects not only the respiratory system, but also other vital organs in the body. We report here a child with an atraumatic splenic rupture as the initial and only manifestation of COVID-19.

**Case presentation:**

A 13-year-old boy with clinical signs of acute abdomen, left-sided abdominal pain, and hemodynamic instability was admitted to the PICU in critical condition. His parents denied any trauma had occurred. In addition to imaging tests, a nasopharyngeal swab was taken for COVID-19 testing, which was positive. The thoracic CT scan was normal, whereas the abdominal CT scan revealed hemoperitoneum, splenic rupture, and free fluid in the abdomen.

**Conclusions:**

The spleen is one of the organs targeted by the SARS-CoV-2. Splenic rupture, a potentially fatal and uncommon complication of COVID-19, can be the first and only clinical manifestation of the disease in children. All pediatricians should be aware of the possibility of atraumatic splenic rupture in children with COVID-19.

## Background

Coronavirus disease 2019 (COVID-19) in children is usually mild. Rarely they can be severely affected with respiratory failure and multisystem involvement [1]. Recent studies have revealed that severe acute respiratory syndrome coronavirus 2 (SARS-CoV-2) affects not only the respiratory system but also other vital organs in the body [2]. SARS-CoV-2 tropism for the spleen has already been demonstrated in patients infected with the virus. It appears to have a direct effect on the spleen and lymph nodes, resulting in severe tissue damage [3–8]. Infections are a significant etiological factor in atraumatic splenic rupture [9–19]. Recently, there have been a few reports of atraumatic splenic rupture in adults with COVID-19, implying that SARS-CoV-2, like other infections, could be the cause [7, 8, 20, 21].

We present the first case of atraumatic splenic rupture in a child, most likely caused by COVID-19. The spleen is a highly vascular organ that filters approximately 10–15% of total blood volume per minute and significant blood loss can occur after rupture from either the parenchyma or the splenic vascular supply. The most common cause of splenic rupture is trauma, while atraumatic splenic rupture is uncommon [12]. Clinical signs of early shock appear if intra-abdominal bleeding exceeds 5–10% of blood volume. Splenic rupture is typically characterized by left-sided abdominal pain and hemodynamic instability. In approximately half of the cases, there is left shoulder-tip pain (Kehr’s sign), which is caused by intraperitoneal blood, causing diaphragmatic irritation [11]. Splenic rupture is generally not considered in the differential diagnosis of abdominal pain, in the absence of trauma. A high index of suspicion for atraumatic splenic rupture is important not only because the condition is uncommon, but also because a delayed diagnosis and treatment of splenic rupture can be life-threatening.

## Case presentation

A 13-year-old boy was admitted to Pediatric Intensive Care Unit (PICU) in a critical situation. After waking up in the morning, he complained of left-sided abdominal pain, nausea, and vomiting. Over the hours, the pain was intermittent and increasing in intensity.

At admission, the patient looked pale, in a forced sitting position. On physical examination, his abdomen was tender in all quadrants with left upper quadrant pain rated as 10 out of 10 in intensity. The pain was described as very strong and increased if he laid down, thus requiring intravenous opioids. Upon examination, heart rate was 92 bpm along with low blood pressure 82/42 mmHg. Diminished breath sounds at the lung bases were noted, most likely due to limited excursions of the chest due to pain. There was no fever (temperature 36.5 C). There was no rash, nor lymphadenopathy. No hematomas or bruises were observed. After intravenous opioids and liquid administration, blood pressure was normalized.

The patient and his father denied any history of trauma. They insisted the child had been totally healthy up until that morning. The child had no family history of coagulopathies, autoimmune diseases, or malignancies. According to his family, there were no bowel abnormalities; use of thrombolytic or anticoagulant drugs.

Several laboratory and imaging examinations were performed immediately. Given the relatively large number of COVID-19 patients during this period, our main differential diagnoses were either a splenic rupture or a splenic artery thrombosis, due to COVID-19. Therefore, a nasopharyngeal swab specimen was collected for COVID-19 testing.

**Fig. 1:**
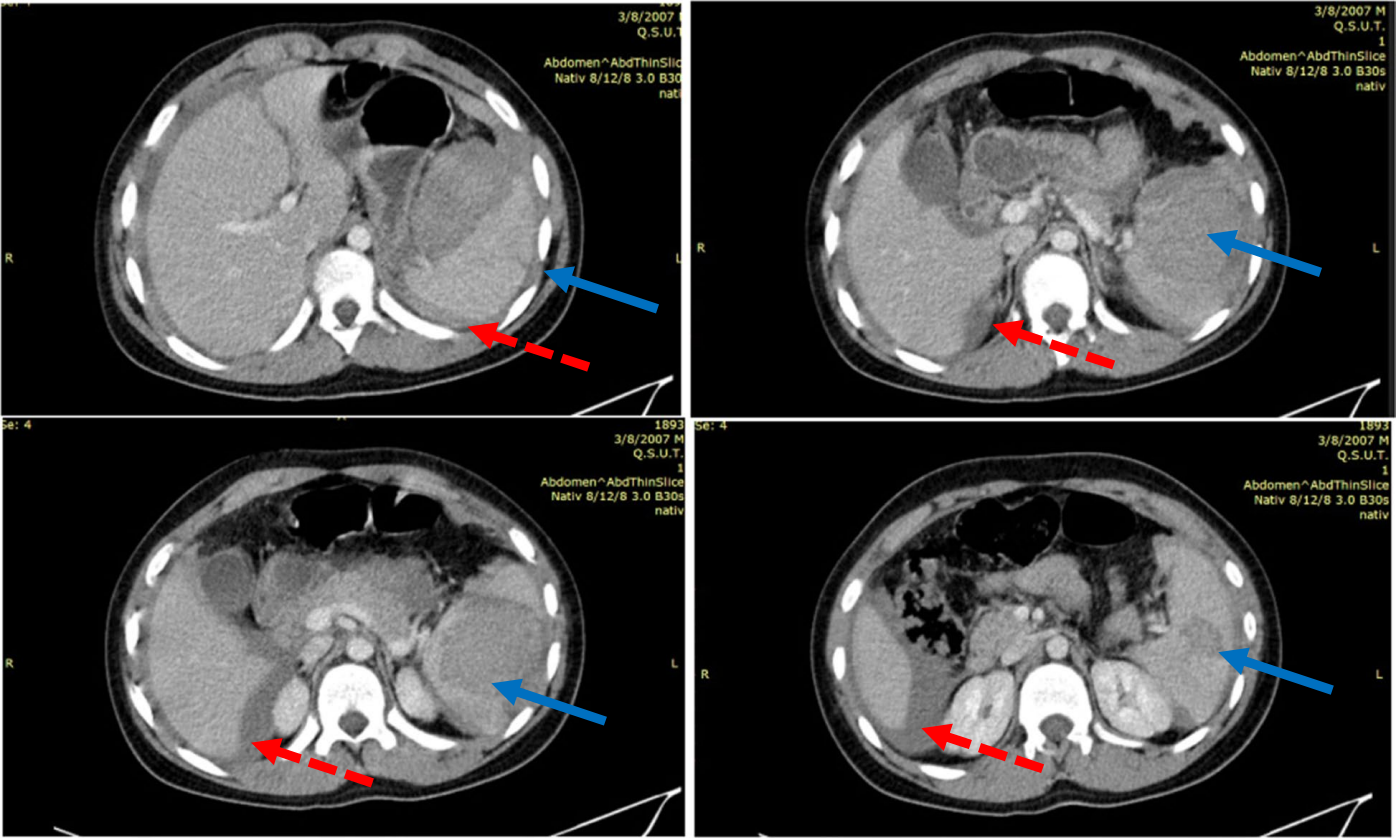
Abdominal CT: There is an intraparenchymal hematoma/laceration measuring > 6 cm which extends to the splenic hilum. (blue solid line) Multiple splenic lacerations were noted inferiorly. No active bleeding on delayed phases. An associated large amount of high density free intraperitoneal fluid/hemoperitoneum around the spleen and liver. (red square dot line) No bone fractures or injuries to other organs

The upright abdominal radiograph showed no abnormalities. Abdominal ultrasound revealed free fluid in the abdomen, but without any clear suspicion, so an emergent Computed tomography (CT) with contrast of the chest and abdomen was carried out. The thoracic CT scan was normal. Abdominal CT (Fig. 1) revealed hemoperitoneum with splenic laceration.

**Fig.2:**
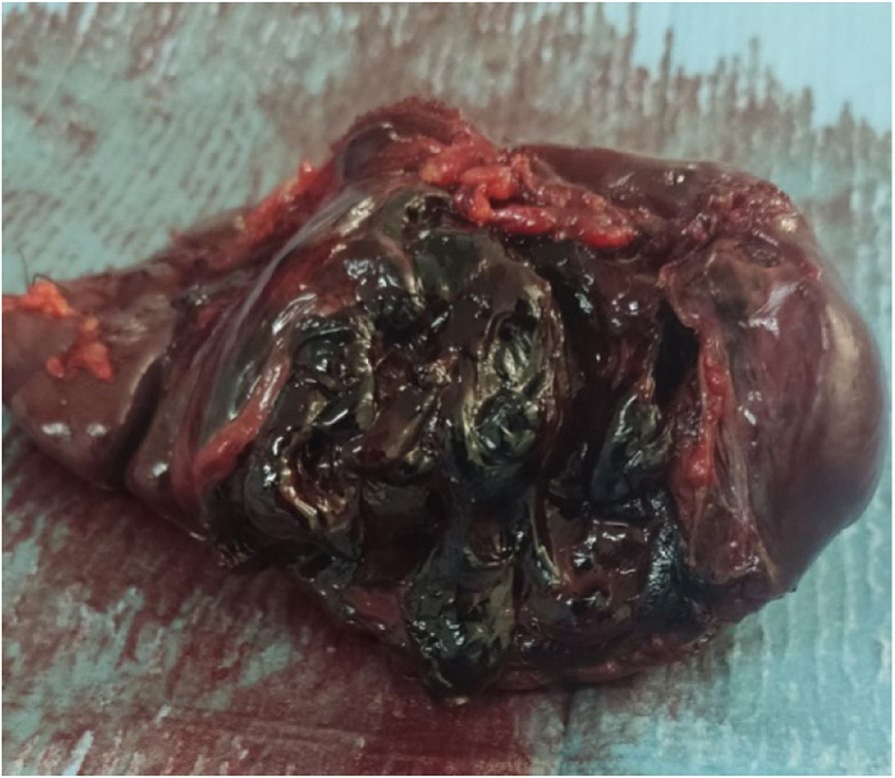
Appearance of the spleen after surgery. In the macroscopic description, the spleen’s measurements were 15 × 9x4 cm (enlarged for his age). Hemorrhagic and necrotic areas of about 8 cm are observed on the hilus. In the microscopic examination, cyclical changes of the type of hemorrhage, necrosis, and vasal congestion were observed

Since the hemoglobin, hematocrit, and patient’s blood pressure were normal, with no active bleeding on CT, the splenic injury was initially managed conservatively. Twelve hours after presentation, a decrease in hemoglobin (Hb = 8.1 g/dL) and hematocrit (HCT = 25.6%) was noted and the patient’s blood pressure started dropping. He received 1 Unit of blood and the decision to proceed to surgery was made. During the operation, it was observed that the patient had plenty of blood in the abdominal cavity. Laceration of the splenic hilum and a large perisplenic hematoma (Fig. 2) was noted and splenectomy was performed. Two additional Units of blood were transfused intraoperatively.

Human immunodeficiency virus (HIV) test, IgM and IgG antibodies for Epstein Barr virus (EBV) and cytomegalovirus (CMV) were negative. The polymerase chain reaction (PCR) was positive for COVID-19, making us think that the splenic rupture could be a consequence of COVID-19. The child’s clinical condition was stable after surgery. He was discharged, without further problems during the follow-up.

## Discussion and conclusions

Splenic rupture is most commonly caused by trauma; however, it can occur without any obvious trauma, which is known as atraumatic or spontaneous splenic rupture, with a reported frequency of less than 1% [22, 23]. Pathologies leading to atraumatic splenic rupture include hematological malignancies, infections, inflammatory diseases and drugs [11, 12, 17]. A literature review by Renzulli et al. showed out that hematological malignancies are the most frequently reported causes of atraumatic splenic rupture with 30% of cases, followed by infections (27%) and inflammatory disease/non-infectious disorders (eg, acute and chronic pancreatitis) with 20% [17]. Atraumatic splenic rupture may occur in a wide age range, from teenagers and young people (particularly from infectious causes) to the elderly and determining the etiology is often challenging. To our knowledge, this is the first case report of atraumatic splenic rupture probably due to COVID-19 in children. There are other cases with atraumatic splenic rupture due to COVID -19, reported in adults [7, 8, 20, 21].

The SARS-CoV-2 virus causes a wide range of disease severity in children [24, 25]. In addition to the respiratory system, it may affect the gastrointestinal tract and other organs [26, 27]. The spleen is one of the organs directly targeted by the virus and according to recent studies, patients with COVID-19 who have left-sided abdominal pain should be evaluated for splenic artery thrombosis and splenic infarction [9, 10, 25, 28, 29]. In their study, Yao Xiaohong et al. suggested that the number of lymphocytes in the spleen was significantly reduced [30]. Hemorrhagic necrosis was present in the spleens of all confirmed SARS studies [26]. Other pathological changes found in our case and reported in all adult cases with atraumatic splenic rupture included thrombosis in a splenic artery as well as rupture and bleeding of a subcapsular hematoma [7, 8, 20, 21].

A spontaneous splenic rupture is a well-known complication of primary EBV infection, but other viruses are also described in literature [9–19]. In a systematic review of 845 patients with an atraumatic splenic rupture, 14.8% were caused by a viral infection, most commonly EBV (74.5% of cases), CMV infection with 9.5% of cases and HIV with 5.8% of cases [17]. The mechanism for the spontaneous hematoma and the splenic rupture is not yet fully clear. Infectious diseases can induce increased intrasplenic tension caused by cellular hyperplasia and vascular occlusion [18]. Similar to these viruses, SARS-CoV-2 could affect the spleen and cause an atraumatic rupture.

Any child with confirmed or suspected COVID-19, in whom abdominal pain develops and extends to the left supraclavicular region (the left shoulder), should be highly suspected of having splenic rupture. Acute abdominal pain can be caused by a wide range of conditions, some of which are life-threatening making the diagnosis challenging. During the COVID-19 pandemic, the diagnostic uncertainty for children with abdominal pain has increased. Gastrointestinal symptoms, which mimic an acute abdomen, can be the primary manifestation of SARS-CoV-2 infection. On the other side, it is well accepted that the differential diagnosis of abdominal pain should include pediatric inflammatory multisystem syndrome (MIS-C), which manifests as abdominal pain in 50% of affected children. The abdominal pain in MIS-C can be so severe that patients were misdiagnosed with peritonitis or another surgical abdominal condition in many cases reported in the literature [31–33]. A thorough history and physical exam can usually narrow the differential diagnosis. The main differential diagnoses in our case were splenic rupture and splenic artery thrombosis. Chest and abdominal CT with contrast along with additional laboratory examinations, helped us exclude other differential diagnosis as pneumonia, pancreatitis, malignancies, etc.

Splenic rupture is characterized by nonspecific abdominal soreness in the left upper quadrant with or without distention, syncope, and a rapid drop in blood pressure, as in our case. It is usually diagnosed later and is a major challenge for radiologists. In cases where the patient is hemodynamically stable and can safely undergo the diagnostic imaging procedure, a CT allows confirmation of the presumptive diagnosis [34, 35]. If the splenic injury is minor, conservative therapy consisting of fluids, with or without blood transfusion(s), and intensive care unit (ICU) admission for close monitoring may be sufficient [34, 35]. When conservative management fails to achieve hemodynamic stabilization, splenectomy may be indicated [34].

Four adult cases of splenic rupture caused by SARS-COV-2 infection are described in the literature. Three of these patients required an emergency splenectomy due to unresponsive hemorrhagic shock, as was the case in our case [9–11]. Only one case underwent a splenic artery embolization procedure to stop the hemorrhage [7]. Regardless of etiology, suspicion, and immediate management of atraumatic splenic rupture according to the degree of splenic injury are crucial [36].

In conclusion we believe that the spleen is one of the organs targeted by SARS-CoV-2. Splenic rupture, a life-threatening and uncommon complication of COVID-19, can be the initial and only clinical sign of the disease in children. All pediatricians should be aware of the rare, however possible occurrence of splenic rupture in children with COVID-19. Unlike in adult cases (with respiratory symptoms in addition to the atraumatic splenic rupture), the atraumatic splenic rupture was the first and only manifestation of COVID-19 in our case report [7, 10, 11].

## Data Availability

There are no more specific data that could be shared.

## Abbreviations

COVID-19: Coronavirus disease 2019
SARS-CoV-2: Severe acute respiratory syndrome coronavirus 2
PICU: Pediatric Intensive Care Unit
CT: Computed tomography
Hb: Hemoglobin
HCT: Hematocrit
HIV: Human immunodeficiency virus
EBV: Epstein Barr virus
CMV: Cytomegalovirus
PCR: Polymerase chain reaction
ICU: Intensive care unit
MIS-C: Multisystem inflammatory syndrome children
